# Increase in peripheral blood mononuclear cell Toll-like receptor 2/3 expression and reactivity to their ligands in a cohort of patients with wet age-related macular degeneration

**Published:** 2013-08-06

**Authors:** Yi Zhu, Liang Liang, Dan Qian, Hongsong Yu, Peizeng Yang, Bo Lei, Hui Peng

**Affiliations:** Department of Ophthalmology, the First Affiliated Hospital of Chongqing Medical University, Chongqing Key Laboratory of Ophthalmology, Chongqing Eye Institute, Chongqing, China

## Abstract

**Purpose:**

To investigate Toll-like receptor (TLR) expression and reactivity in patients with the wet form age-related macular degeneration (AMD).

**Methods:**

Blood samples were collected from 25 patients with wet AMD and 25 age-matched healthy controls. Peripheral blood mononuclear cells (PBMCs) were isolated with Ficoll-Hypaque density gradient centrifugation. Expression of TLR1 to TLR10 mRNAs in PBMCs from 15 patients with wet AMD and 15 controls was assessed with real-time PCR. TLR2 and TLR3 protein levels in PBMCs from six patients with wet AMD and six controls were measured with flow cytometry. After PBMCs were stimulated with peptidoglycan (PGN) and poly(I:C), the specific ligands of TLR2 and TLR3, cytokines interleukin-6 (IL-6), IL-8, VEGF, and monocyte chemoattractant protein-1 (MCP-1) production in 11 patients with wet AMD and 11 controls were assessed.

**Results:**

TLR2 and TLR3 mRNA and protein expression in the PBMCs of the patients with wet AMD was significantly higher than that in the controls. However, the difference in TLR1 and TLR4–10 mRNA expression between the two groups was not significant. The PBMCs of the patients with wet AMD produced more IL-6 and IL-8 proteins than the controls in response to PGN, a ligand for TLR2, and more IL-6 protein than the controls in response to poly(I:C), the ligand for TLR3. However, there was no significant difference in vascular endothelial growth factor and monocyte chemoattractant protein-1 production between the wet AMD group and the control group when the PBMCs were stimulated with PGN or poly(I:C).

**Conclusions:**

Our data suggested that upregulation of TLR2 and TLR3 may be associated with the pathogenesis of wet AMD.

## Introduction

Toll-like receptors (TLRs) are a family of innate immune system receptors that initiate signals in response to diverse pathogen-associated molecular patterns in the innate immune system [1,2]. Ten functional TLRs (TLR1 to TLR10) have been identified in humans. TLRs are expressed in various mammalian immune-related cells such as mast calls, macrophages, monocytes, and dendritic cells [3,4]. Each TLR recognizes different microbial components as ligands. For example, TLR2 recognizes peptidoglycans (PGNs) in the cell wall of Gram-positive bacteria [5], and TLR3 recognizes poly(I:C) produced during virus replication [6]. On the binding of specific ligands, TLRs trigger a signaling cascade and result in cytokine production, which drives an inflammatory response and activates the adaptive immune system [7].

Age-related macular degeneration (AMD) is the leading cause of blindness in the elderly all over the world [8]. It is believed that inflammation plays an important role in AMD pathogenesis [9,10]. A mutation of the complement factor H gene, which is involved in a component of the immune system that regulates inflammation, has been linked to AMD [11]. However, mechanisms initiating and perpetuating inﬂammation in AMD remain to be elucidated. Further investigations concerning how the immune system dysfunction causes AMD will not only elucidate the underlying mechanisms but also lead to novel therapeutic strategies.

Multiple genetic and environmental factors [12], such as innate immunity and oxidative stress, are associated with AMD. Human retinal pigment epithelium (RPE) cells play a principal role in immune defense. The RPE expresses TLR1–7 and 9–10, and its dysfunction is crucial for the clinical changes seen in AMD [13]. Treatment of RPE with poly(I:C) resulted in increased production of proinflammatory cytokines and chemokines [14]. Kleinman and colleagues found that activation of TLR3 by poly(I:C) induced RPE death, suggesting the possible involvement of TLR3 activation in AMD [15]. Similarly, TLR4 activation leads to increased cell death during oxidative injury or photoreceptor protection from oxidative stress depending on the timing of TLR4 signaling [16]. *Chlamydia pneumoniae* LPS (cLPS) presents a positive correlation with the progression of wet AMD [17]. Characterized by choroidal neovascularization (CNV), wet AMD is the most severe form of AMD [18]. Vascular endothelial growth factor (VEGF) is the most important factor in the development of CNV. TLR2 is essential for VEGF and interleukin-6 (IL-6) production from RPE cells in vitro and for *C. pneumoniae* antigen-induced CNV in vivo [19]. High concentrations of poly(I:C) increase VEGF secretion in the RPE [15]. In addition, recent reports have highlighted a general TLR3-mediated antiangiogenic effect of small interfering RNAs (siRNAs) in animal models of CNV [20]. These data suggest that TLRs are relevant to the pathogenesis of wet AMD.

To further investigate whether TLRs are associated with wet AMD, we studied TLR1–10 messenger ribonucleic acid (mRNA) expression proﬁles on the peripheral blood mononuclear cells (PBMCs) in a cohort of patients with wet AMD. We found TLR2 and TLR3 mRNA and protein expression in the patients with wet AMD was significantly higher than that in the controls. Furthermore, we measured the production of cytokines from the PBMCs upon activation by TLR2 and TLR3 ligands. We found ligand-mediated IL-6 and IL-8 production was increased in patients with wet AMD.

## Methods

### Subjects

This study was approved by the Ethics Committee of the First Affiliated Hospital of Chongqing Medical University, Chongqing, China (#2011–23). All procedures complied with the Declaration of Helsinki, and informed consent was obtained from all patients with AMD and the controls.

A total of 25 consecutive patients from the Chongqing area were recruited from April to October in 2012. Diagnosis of wet AMD was made based on an international standard [21] by a senior ophthalmologist. Patients with accompanying systemic autoimmune diseases, other immune-related diseases, hypertension, diabetes, nephropathy, hyperlipidemia, coronary heart disease, heart failure or renal failure, or a history of cancer within the past 5 years, or patients who were cognitively impaired or unable to provide written informed consent were excluded from the study. Patients with other ocular conditions such as diabetic retinopathy, cataract, polypoidal vasculopathy, and ocular inflammatory diseases were also excluded. Twenty-five consecutive age-matched healthy volunteers from the Chongqing area were included as controls. People with accompanying systemic disease and oculopathy were excluded from the study. All the patients underwent routine eye examinations and fundus photograph, optical coherence tomography, fundus fluorescein angiography, and indocyanine green angiography examinations.

### Cell isolation and culture

Heparinized whole blood was obtained from each individual, and PBMCs were prepared within 4 h after blood samples were collected. PBMCs were isolated by Ficoll-Hypaque density gradient centrifugation and were frozen in TRIzol (Invitrogen, Carlsbad, CA) at a concentration of not more than 7×10^6^/ml. The cells were stored at −80 °C or cultured immediately. PBMCs were resuspended at a concentration of 1×10^6^ cells/ml in RPMI-1640 medium (Gibco, Invitrogen), containing L-glutamine (2 mM), penicillin/streptomycin (100 U/ml), and 10% fetal calf serum.

### Real-time quantitative polymerase chain reaction analysis

Total RNA was extracted with TRIzol (Invitrogen) following the manufacturer’s instructions. RNA concentrations were determined with a Nano instrument (NanoDrop Technologies, Wilmington, DE). The first-strand cDNA was synthesized for each RNA sample using the Superscript III Reverse Transcriptase system (Invitrogen). Real-time quantitative PCR was performed on the iCycler (Biorad, Hercules, CA) using the Quanti Tect SYBR Green PCR kit (Applied Biosystems, Foster City, CA). The forward and reverse primers for β-actin were designed using Primer Premier software (Premier Biosoft International, Palo Alto, CA). The sequences of the PCR primer pairs are shown in Table 1. The human β-actin gene was used as an endogenous control for sample normalization. For each sample, the relative abundance of target mRNA was calculated from the C_Δt_ values for the target and endogenous reference gene β-actin by using the 2^-ΔΔCt^ cycle threshold method.

**Table 1:** List of all primer sequences.

Gene	Forward primer	Reverse primer
β-actin:	GGATGCAGAAGGAGATCACTG	CGATCCACACGGAGT ACTTG
TLR1	GGATGCAGAAGGAGATCACTG	TTTCAAAAACCG TGTCTGTTAAGAGA
TLR2	GGAGGCTGCATATTCCAAGG	GCCAGGCATCCTCACAGG
TLR3	CAGTGTCTGGTACACG CATGGA	TTTCAAAAACCGTGTCTGTTAAGAGT
TLR4	AGTTTCCTGCAATGGATCAAGG	CTGCTTATCTGAAGGTGTTGCA
TLR5	GGCTTAATCACACCAATGTCACTATAG	TTAAGACTTCCTCTTCATCACAA CCTT
TLR6	CATCCTATTGTGAGTTTCAGGCAT	GCTTCATAGCACTCAATCCCAA
TLR7	TGGAAATTGCCCTCGTTGTT	GTCAGCGCATCAAAAGCATT
TLR8	AGCGGATCTGTAAGAGCTCCATC	CCGTGAATCATTTTCAGTCAAGAC
TLR9	AGCGGATCTGTAAGAGCTCCATC	CCGTGAATCATTTTCAGTCAAGA
TLR10	AAGAAAGGTTCCCGCAGACTT	TGTTATGGCATAGAATCAA AACTCTCA

### Cytokine analysis

Supernatants collected from cell culture were tested with enzyme-linked immunosorbent assay (ELISA). The protein concentrations of IL-6, IL-8, monocyte chemoattractant protein-1 (MCP-1), and VEGF were determined using human ELISA development kits (R&D Systems, Minneapolis, MN) according to the manufacturer’s instructions with detection limits of 9.4 pg/ml, 15.6 pg/ml, 31.2 pg/ml, and 5.0 pg/ml, respectively. For stimulation of PBMCs TLR2 or TLR3, whole blood from 11 patients with wet AMD and 11 healthy controls was analyzed. PBMC cytokine production in response to the TLR2 ligand (PGN, 5 μg/ml; Fluka, Buchs, Switzerland) or the TLR3 ligand (poly(I:C), 50 μg/ml; Fluka) was measured at 48 h after ligand stimulation.

### Flow cytometric analysis

To detect TLR2 and TLR3 protein expression, PBMCs were incubated for 30 min at 4 °C with antihuman CD282 (TLR2) or CD283 (TLR3) PE (eBioscience, San Diego, CA) and mouse IgG2a/IgG21 K Isotype Control PE (eBioscience). Cells were washed with phosphate buffered saline (PBS; KCl 0.2 g/l, KH_2_PO_4_ 0.2 g/l, NaCl 8.0 g/l, Na_2_HPO_4_ 1.15 g/l) solution and resuspended in cold PBS solution for flow cytometric analysis.

### Statistical analysis

The analysis was performed using SPSS 17.0 (SPSS Inc., Chicago, IL). Data are expressed as the mean±standard deviation (SD) or standard error of the mean (SEM). Student *t* test and Mann–Whitney tests were used to compare the data for the patients with wet AMD and the healthy controls. Differences reaching p<0.05 were considered statistically signiﬁcant.

## Results

### Expression of TLR mRNAs from peripheral blood mononuclear cells in patients with wet AMD

**Figure 1:**
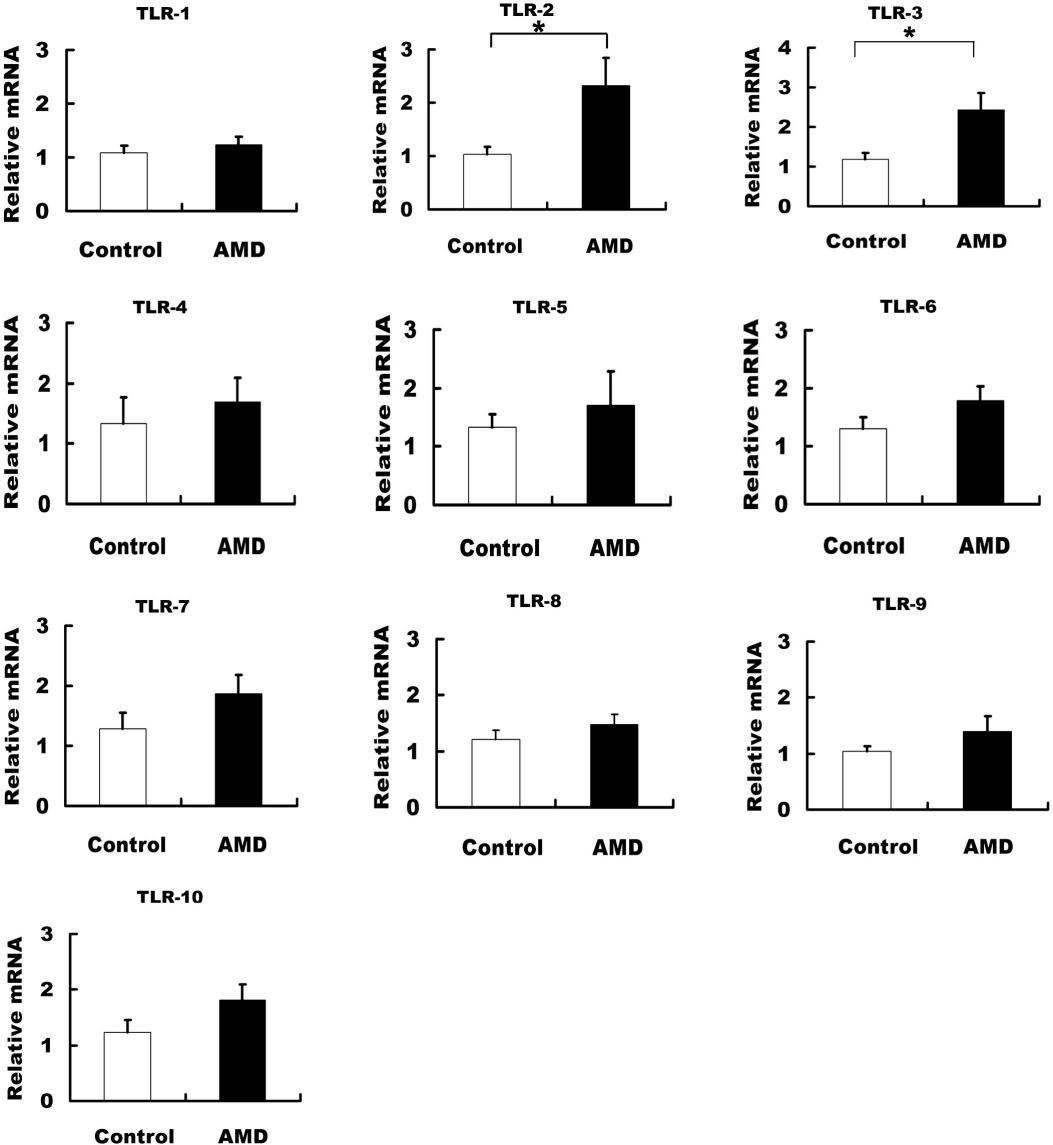
Expressions of toll-like receptor 1 (TLR1)-10 mRNA in peripheral blood mononuclear cells (PBMCs) of wet age-related macular degeneration (AMD) patients. TLR2 and TLR3 mRNA expressions were higher in the wet AMD group (n=15) than in the control group (n=15; t-test, * p<0.05).

Fifteen patients with wet AMD and 15 controls were studied to determine the expression levels of the TLR1–10 transcripts. Freshly isolated PBMCs were used for real-time PCR. As shown in Figure 1, the TLR2 and TLR3 mRNA levels of the patients with wet AMD were significantly higher than those of the controls (Figure 1 , p<0.05). However, the differences in the mRNA levels of other TLRs (TLR1, TLR4–10) between the wet AMD group and the control group were not significant.

### Cell surface TLR2 and TLR3 protein expression in PBMCs of wet AMD patients

**Figure 2:**
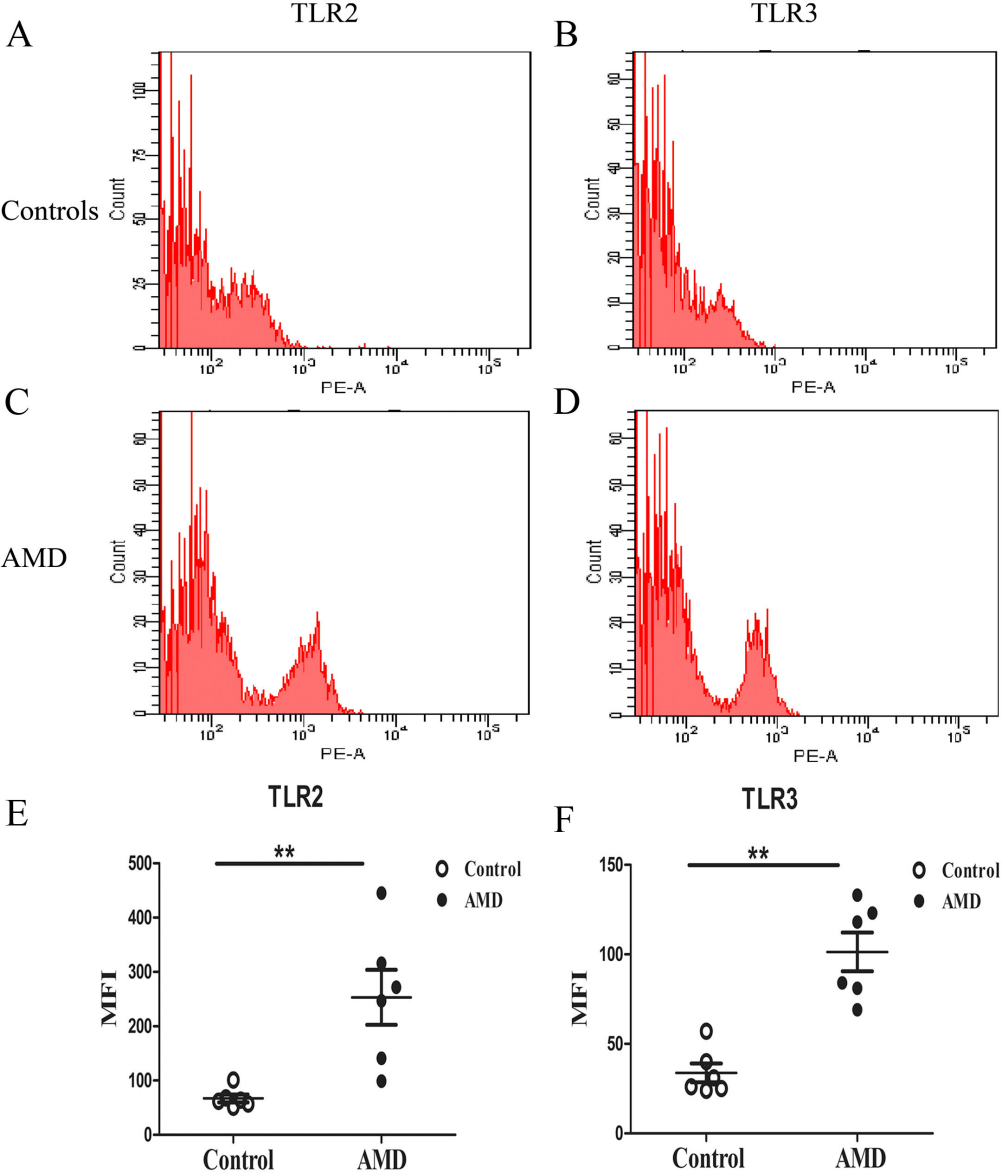
Toll-like receptor 2 (TLR2) and TLR3 protein expressions in peripheral blood mononuclear cells (PBMCs) of wet age-related macular degeneration (AMD) patients compared to healthy control. Representative TLR2 and TLR3 protein expressions of PBMC from patients (n=6; **A**, **C**) and healthy controls (n=6; **B**, **D**) measured with flow cytometer (FCM). Expressions of TLR2, TLR3 protein were higher in PBMCs of AMD group than in the control group (**E**, **F**). Mann-Whitney test, **p<0.01.

Since the mRNA levels of TLR2 and TLR3 in the PBMCs of the patients with wet AMD were significantly higher than those in the controls, we detected PBMC TLR2 and TLR3 protein expression. Samples from six patients with wet AMD and six controls were analyzed. In agreement with the mRNA results, the PBMC TLR2 and TLR3 protein levels were significantly higher in the patients with wet AMD than in the controls (Figure 2, p<0.05).

### Increased IL-6 and IL-8 expression and enhanced TLR2 and TLR3 responses to ligand stimulation of PBMCs from wet AMD patients

**Figure 3:**
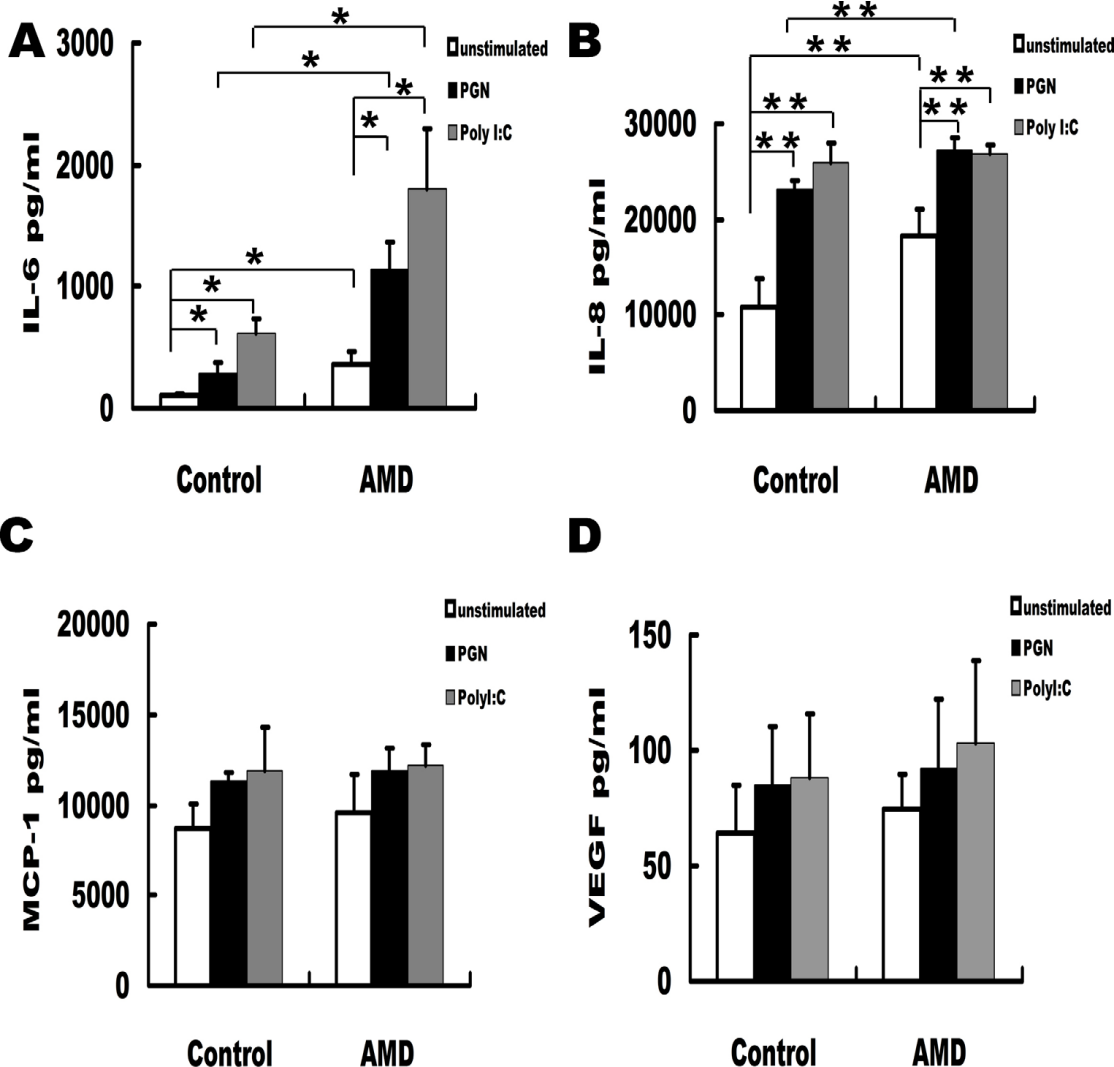
Increasing of peripheral blood mononuclear cell (PBMC) toll-like receptor 2 (TLR2) and TLR3 expressions and their reactivity to TLR ligands in wet age-related macular degeneration (AMD) patients. PBMCs were stimulated with PGN (5 μg/ml) and PolyI;C (50 μg/ml) respectively for 48 h. The PBMCs of the wet AMD patients (n=11) produced much higher IL-6 and IL-8 than the controls (n=11) in response to PGN, and much higher IL-6 than the controls in response to PolyI:C (**A**, **B**). The productions of MCP-1and VEGF showed no significant difference between the two groups (**C**, **D**) Mann–Whitney test, *p<0.05, **p<0.01.

The increased expression of TLR2/3 in the PBMCs of patients with wet AMD raises the possibility that both TLRs could be involved in this disease through interaction with their ligands, PGN and poly(I:C). Further experiments were therefore performed to investigate the effects of their interaction on the production of IL-6, IL-8, MCP-1, and VEGF in PBMCs, which have been reported to have signiﬁcantly higher concentrations in the aqueous humor of wet AMD and are involved in the pathogenesis of wet AMD [22-25]. We found that IL-6 (Figure 3A) and IL-8 (Figure 3B) expression in the PBMCs of the patients with wet AMD was significantly higher than that of the controls. PGN and poly(I:C) significantly increased IL-6 and IL-8 production in the PBMCs from patients with wet AMD and the controls. However, the PBMCs of the patients with wet AMD produced significantly more IL-6 and IL-8 than the controls in response to PGN, and only significantly more IL-6 than the controls in response to poly(I:C). There was no significant difference between the patients with wet AMD and the controls concerning the production of MCP-1 and VEGF in PBMCs with or without PGN or poly(I:C) stimulation (Figure 3C,D).

## Discussion

In the present study, we observed significantly increased mRNA and protein expression of TLR2 and TLR3 in PBMCs from patients with wet AMD compared with the healthy controls. The PBMCs of the patients with wet AMD produced significantly more IL-6 and IL-8 than the controls in response to the TLR2 ligand (PGN), and significantly more IL-6 than the controls in response to the TLR3 ligand (poly(I:C)). MCP-1 and VEGF production by the PBMCs showed no significant difference between the two groups.

Recent studies suggested that pathogenesis of AMD is related to chronic inﬂammatory processes. Increasing evidence has demonstrated that the immune system could play a central role in AMD. TLRs are expressed in ocular tissues and play an important role in defending against various pathogens in the eye [26]. To test whether TLRs in the circulating blood are associated with AMD, we studied the mRNA expression of TLR1–10 in the PBMCs of patients with wet AMD. We found that the mRNA expression of TLR2 and TLR3 in patients with wet AMD was significantly higher than that in the controls. In agreement with the mRNA expression, the protein levels of TLR2 and TLR3 in patients with wet AMD were also significantly higher than those in the controls. Our data demonstrated that the circulating TLR2 and TLR3 expression was significantly higher in the patients with wet AMD. Enhanced circulating TLR2 and TLR3 expression, at the mRNA and protein levels, may be a nonspecific biomarker of wet AMD.

A close connection between TLR3 and wet AMD is supported by clinical and experimental observations. It has been reported that CNV membranes from eight patients with wet AMD all expressed TLR3 [27]. Activation of TLR3 by poly(I:C) in the RPE increases the production of VEGF, which promotes the development of CNV [15]. However, other studies showed activation of TLR3 suppressed experimental CNV [20,28,29]. The suppression of CNV in this model has previously been explained as a VEGF-A-independent effect mediated by the toxic effects of TLR3 activation on endothelial cells [20]. Meanwhile, the physiologic relevance of VEGF secretion in RPE under high concentrations of poly(I:C) has yet to be confirmed [15]. More investigations are needed to reveal the detailed mechanisms of TLR3 in the pathogenesis of CNV. It was reported recently that *C. pneumoniae* triggered inflammatory responses in the eye and promoted experimental CNV in a TLR2-dependent manner [19]. Our results support the notion that the TLR2 signaling pathway may play an important role in wet AMD pathogenesis. TLR2 is usually associated with TLR1 or TLR6 to form a heterodimer and recognize peptideglycans in the cell wall of Gram-positive bacteria. However, in our study we found only TLR2 expression in PBMCs was significantly increased in patients with wet AMD. The PBMCs of the patients with wet AMD experienced an increase in TLR1 and TLR6 expression compared to the PBMCs of the control group, but there was no significant difference between the two groups. This may be due to the small sample size of our study. More studies are needed.

Intraocular concentrations of IL-6, IL-8, MCP-1, and VEGF are significantly associated with the pathogenesis of wet AMD [22-25]. To test whether the upregulated TLR2 and TLR3 are correlated with the expression of IL-6, IL-8, MCP-1, and VEGF, we measured the expression of these cytokines in the supernatants of PBMCs in patients with wet AMD and controls without stimulation. Consistent with previous studies, we found that the IL-6 and IL-8 production was significantly higher in patients with wet AMD.

To further understand whether the functions of TLR2 and TLR3 in the PBMCs of patients with wet AMD are altered, we measured the concentration of IL-6, IL-8, MCP-1, and VEGF in the supernatants of PBMCs after stimulation with their ligands, PGN and poly(I:C). The results showed that PGN and poly(I:C) significantly increased IL-6 and IL-8 production by PBMCs from the patients with wet AMD and the controls. However, IL-6 and IL-8 production by PBMCs stimulated with PGN in the patients with wet AMD was significantly higher than that in the controls; IL-6 production by PBMCs stimulated by poly(I:C) in patients with wet AMD was significantly higher than that in controls. There was no difference between the patients with wet AMD and controls concerning the production of MCP-1 and VEGF with or without PGN or poly(I:C) stimulation. Our results are, by and large, consistent with earlier studies. It has been reported that activation of TLR3 in PBMCs of patients with diabetes can increase the expression of IL-6 [30]. It also has been reported that the expression of TLR3 was significantly increased in the PBMCs of patients with enthesitis-related arthritis and the interaction of TLR3 with poly(I:C) significantly increased the production of IL-6 and IL-8 [31]. *C.* *pneumonia*e antigen-mediated IL-6 production was markedly reduced in TLR2 knockout mice [19]. An experiment concerning septic arthritis reported that PGN increased IL-8 production by the PBMCs of patients with septic arthritis [32]. Many studies concerning the expression of VEGF in the eye have been conducted [33,34]; anti-VEGF therapy has been proved to be an effective and safe way to treat wet AMD [35,36]. However, VEGF expression in the peripheral blood has never been reported. In our study, we found that VEGF production by the PBMCs of patients with wet AMD and controls was similar. This result suggested that VEGF expressed by PBMCs might not be involved in the pathogenesis of wet AMD. All these data indicated that the reactivity of TLR2 and TLR3 in patients with wet AMD was significantly increased. The increased activity of TLR2 and TLR3 may be an indicator of an inflammatory milieu in patients with wet AMD.

In conclusion, we found that the expression and reactivity of TLR2/3 in the PBMCs of patients with wet AMD were significantly increased. These results suggested that TLR2/3 may play a role in the pathogenesis of wet AMD. Further studies are needed to elucidate the detailed roles of TLR2 and TLR3 in the development of wet AMD.
